# Successive tendon injury in an *in vivo* rat overload model induces early damage and acute healing responses

**DOI:** 10.3389/fbioe.2024.1327094

**Published:** 2024-03-07

**Authors:** Pooja H. Chainani, Maria Buzo Mena, Diana Yeritsyan, Daniela Caro, Kaveh Momenzadeh, Jenna L. Galloway, Joseph P. DeAngelis, Arun J. Ramappa, Ara Nazarian

**Affiliations:** ^1^ Musculoskeletal Translational Innovation Initiative, Carl J. Shapiro Department of Orthopaedic Surgery, Beth Israel Deaconess Medical Center, Harvard Medical School, Boston, MA, United States; ^2^ Department of Mechanical Engineering, Boston University, Boston, MA, United States; ^3^ Center for Regenerative Medicine, Massachusetts General Hospital, Harvard Medical School, Boston, MA, United States; ^4^ Carl J. Shapiro Department of Orthopaedic Surgery, Beth Israel Deaconess Medical Center, Harvard Medical School, Boston, MA, United States; ^5^ Department of Orthopaedic Surgery, Yerevan State Medical University, Yerevan, Armenia

**Keywords:** tendinopathy, mechanobiology, fatigue, tendon overload, *in vivo*, acute healing, injury

## Abstract

**Introduction:** Tendinopathy is a degenerative condition resulting from tendons experiencing abnormal levels of multi-scale damage over time, impairing their ability to repair. However, the damage markers associated with the initiation of tendinopathy are poorly understood, as the disease is largely characterized by end-stage clinical phenotypes. Thus, this study aimed to evaluate the acute tendon responses to successive fatigue bouts of tendon overload using an in vivo passive ankle dorsiflexion system.

**Methods:** Sprague Dawley female rats underwent fatigue overloading to their Achilles tendons for 1, 2, or 3 loading bouts, with two days of rest in between each bout. Mechanical, structural, and biological assays were performed on tendon samples to evaluate the innate acute healing response to overload injuries.

**Results:** Here, we show that fatigue overloading significantly reduces *in vivo* functional and mechanical properties, with reductions in hysteresis, peak stress, and loading and unloading moduli. Multi-scale structural damage on cellular, fibril, and fiber levels demonstrated accumulated micro-damage that may have induced a reparative response to successive loading bouts. The acute healing response resulted in alterations in matrix turnover and early inflammatory upregulations associated with matrix remodeling and acute responses to injuries.

**Discussion:** This work demonstrates accumulated damage and acute changes to the tendon healing response caused by successive bouts of *in vivo* fatigue overloads. These results provide the avenue for future investigations of long-term evaluations of tendon overload in the context of tendinopathy.

## 1 Introduction

Tendons are highly aligned, fibrous tissues connecting muscle to bone and are subjected to substantial amounts of daily loads; the Achilles tendon, the largest in the body, experiences loads up to 12.5 times the body weight during running (Komi et al., 1992; Svensson et al., 2016). Thus, tendons are prone to ruptures and overuse injuries, such as tendinopathies, from their daily continuous stresses and strains. Tendinopathy causes functional impairments, painful symptoms, and decreased quality of life in 30%–50% of the elderly, workplace, and athletic populations (Andarawis-Puri and Flatow, 2011; Kaux et al., 2011; Neviaser et al., 2012; Maffulli et al., 2020). Achilles tendinopathy has a lifetime incidence of 52% for athletes involved in running activities (Tarantino et al., 2023). Clinical cases of tendinopathy present morphological changes (i.e., increased cross-sectional area) as well as diminished mechanical and material properties (Arya and Kulig, 2010; Helland et al., 2013; Wiesinger et al., 2020) Despite its prevalence and substantial healthcare burden, the pathogenesis is largely unknown and clinically characterized in its later stages when the condition is accompanied by pain as well as degenerative symptoms, limiting treatment options.

External mechanical stimuli from physical activity drive tendon homeostasis, adaptation, and repair responses. For example, macroscale loads cause microscopic tensile, compressive, shear, and fluid changes to the ECM due to the hierarchical and viscoelastic properties of the tendon. Native tendon cells (tenocytes) sense and convert mechanical stimuli into cell signals through cell-cell and cell-matrix interactions and respond through mechanotransduction pathways of tissue remodeling and adaptation. Force transmission through the tendon sets off a cascade of biological responses that can strengthen the tendon through matrix synthesis and degradation of damaged collagen and the surrounding ECM (Lipman et al., 2018). However, abnormal loading conditions-such as overload-ultimately trigger a cascade of mechanobiological signals due to deviations from the cellular tensional homeostasis (Pentzold and Wildemann, 2022). Lack of sufficient rest periods between abnormal loading conditions causes an increase in the deposition of mechanically weaker type III collagen, collagen fiber disruption, net degradation of tendon ECM, apoptosis, and inflammation, leaving the tendon susceptible to further injury (Andarawis-Puri et al., 2014; Spiesz et al., 2015; Thorpe et al., 2015; Zamboulis et al., 2020). The accumulation of fatigue damage coupled with insufficient recovery time results in a chronic degenerative cycle resulting from a “failed” tendon healing response, ultimately leading to tendinopathy.

Thus, multiscale investigations of tendon damage are critical to understanding how damage propagates spatially and temporally. *In vivo* animal models of tendon overuse and overload (such as treadmill running, repetitive reaching, climbing, direct tendon loading, and synergist ablation) have been used in such work (Williamson et al., 2021; Bloom et al., 2023). Recently developed *ex vivo* model systems-designed to mimic *in vivo* tendon biomechanics and load the tendon in isolation-demonstrated that cyclically loading tendons to physiological stresses and strains results in temporal inflammatory as well as matrix degradation responses (Benage et al., 2022). Sub-failure tendon changes such as decreased stiffness and collagen disorganization, indicative of early signs of degeneration, have been revealed in cyclic fatigue loading in rat and mouse models (Soslowsky et al., 2000; Soslowsky et al., 2002; Fung et al., 2009; Ng et al., 2011; Andarawis-Puri et al., 2012; Andarawis-Puri et al., 2014). Various overuse studies have reported increased levels of non-tenogenic cell types and rounded cellular morphology. These may activate mechanotransduction pathways, altering matrix turnover and inflammatory pathways, which may play roles in tendon structural and mechanical degeneration (Williamson et al., 2021).

Whereas *in vivo* overuse models have identified several damage markers associated with overuse tendinopathy in a physiologically relevant manner, models cannot measure a tendon’s stresses and strains during fatigue loading or selectively loading the tendon in isolation from connecting tissues. *Ex vivo* dynamic loading models address these limitations and allow for a coupled understanding of the mechanobiological changes associated with specified fatigue loads but in isolation from the body’s natural environment and reactionary responses to injuries. Therefore, the objective of this study was to assess multiscale mechanobiological and structural changes of the Achilles tendon acute healing in response to successive cyclic fatigue loading, using our previously validated system that applies cyclic loads to the rat Achilles tendon while measuring the tendon’s stresses and strains (Williamson et al., 2023). To determine the effects of fatigue loading on tendon sub-failure changes and the subsequent acute healing response, we assessed changes to *in vivo* mechanical and multiscale morphological properties and biological response through gene expression. We hypothesized that successive fatigue loading would result in decreased *in vivo* mechanical properties, collagen micro-damage, cellular infiltration, and a disruption to the innate inflammatory response and collagen and matrix turnover.

## 2 Materials and methods

### 2.1 Experimental design

**FIGURE 1:**
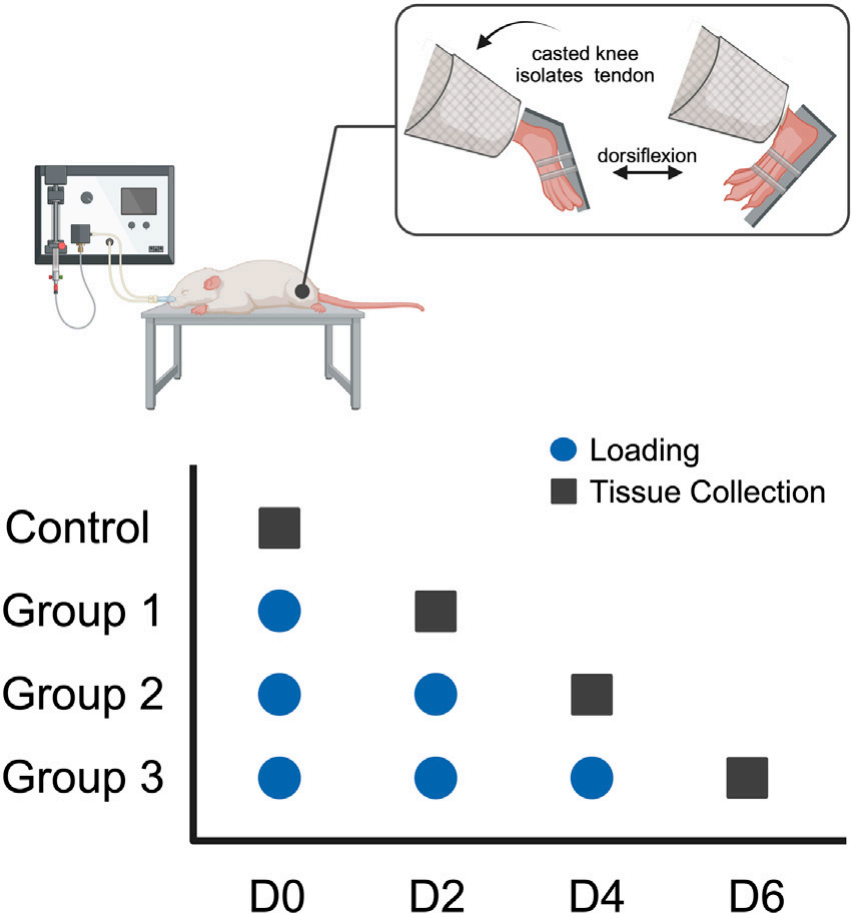
Study design: Thirteen-week-old female Sprague-Dawley rats underwent fatigue loading via a passive ankle dorsiflexion system for 1 (group 1), 2 (group 2), or 3 (group 3) bouts of loading (*n* = 9/group). Loading was performed once a day every 2 days (blue circle) and tendon samples were collected (black square) 2 days following the final loading bout.

All procedures were approved by the Institutional Animal Care and Use Committee (IACUC). In this study, female rats were chosen due to existing studies indicating that women have a greater burden associated with tendon disorders, with greater disabilities and diminished tendon function (Sarver et al., 2017; Pentzold and Wildemann, 2022). Thirteen-week-old female Sprague Dawley rats (*n* = 36) were anesthetized using isoflurane. The left hindlimb of each animal was fixed in full-leg extension using a splint to isolate the Achilles tendon. The foot was attached to an ankle joint actuator that allowed for passive ankle dorsiflexion up to 40° relative to full plantarflexion, and the rat was secured in a prone position in a full-body platform. The ankle was cyclically dorsiflexed to an angle that loaded the tendon for 500 cycles to the exponential region at ∼1 Hz to mimic normal gait, as described in a previously published protocol (Williamson et al., 2023; Chainani et al., 2024). Each rat was subjected to either 0 (control), 1 (group 1), 2 (group 2), or 3 (group 3) bouts of 500 loading cycles (one bout per day, *n* = 9 per group) with 2 days of cage activity between each loading (Figure 1). Mechanical measurements before and after each loading were performed to assess the effect of loading on tendon mechanical properties. Two days after each group’s final loading, the rats were euthanized via CO_2_ inhalation. No loading or mechanical measurements were performed on the control group. The loaded Achilles tendon tissue was harvested for RT-qPCR gene expression analysis (*n* = 5 per group), histological assessment (*n* = 3 per group), and transmission electron microscopy analysis of transverse sections (*n* = 1 per group).

### 2.2 Mechanical data analysis

The torque and angle loading data from five baseline measurements before the loading regimen and five post-loading measurements immediately after the last loading bout were converted to stress and strain using the Achilles tendon moment arm and cross-sectional area obtained from pilot studies. The hysteresis curves were analyzed using a custom-developed MATLAB code (MathWorks, Natick, MA, United States) to calculate percentage differences in hysteresis, peak stress, and loading and unloading moduli between baseline measurements and after the final bout of loading for each group.

### 2.3 Histology

Following euthanasia, tendon tissues were dissected at the calcaneus and proximal to the myotendinous junction and immediately fixed in 10% neutral buffered formalin. The tissue samples were formalin-fixed paraffin-embedded (FFPE) via routine paraffin protocol and sectioned and stained at the BIDMC Histology Core. Sections of 5 µm thickness from each sample were stained with hematoxylin & eosin (H&E) to assess changes in cell shape and size and with Masson’s Trichrome staining to assess collagen structural changes with a standard protocol by the BIDMC Histology Core. Slides were imaged at ×10 using a brightfield full slide scanning microscope (Olympus VS120 Virtual Slide Scanner, Olympus, Japan).

### 2.4 Histological image analysis

Using the open-source image-processing program Fiji (National Institutes of Health, MD, United States), H&E, and Masson’s Trichrome stained images were pre-processed and analyzed to extract cellular morphology and collagen orientation. For H&E stained images, the regions of interest were manually segmented, and a color deconvolution algorithm was performed to isolate the hematoxylin stain, and the cell nuclei were segmented using the Phansalkar thresholding method and watershed segmentation. The circularity, nuclear aspect ratio, area fraction, and cellularity were analyzed for each image. The Masson’s Trichome stained images were manually cropped to the region of interest and converted to greyscale. Using the OrientationJ plugin in Fiji, the collagen orientation was extracted for each image. The circular variance was calculated for each group to measure the spread of orientations per group.

### 2.5 Transmission electron microscopy

Tendon tissues were dissected as previously described for the histological samples. Tissues were immersion fixed in 2% glutaraldehyde (Electron Microscopy Sciences, Hatfield, PA, United States) in 0.1M Sodium Cacodylate (Sigma-Aldrich, Burlington, MA, United States) pH 7.4 for at least 1 h at room temperature and then at 4°C overnight. Tissues were washed with 0.1M Sodium Cacodylate and then post-fixed for 1 h at 4°C in 1% osmium tetroxide (Electron Microscopy Sciences) in 0.1M Sodium Cacodylate. Cells were washed in DI water and incubated in 2% aqueous uranyl acetate (Electron Microscopy Sciences) overnight at 4°C. The following day, tissues were washed with DI water and then dehydrated at 4°C in a graded ethanol series. Tissues were then brought to room temperature and dehydrated with 100% ethanol (Sigma-Aldrich), followed by propylene oxide (Electron Microscopy Sciences). Infiltration with LX112 resin (Ladd Research Industries, Williston, VT, United States) was followed by embedding in flat-bottom Beem capsules (Electron Microscopy Sciences). The resulting blocks were sectioned using a Leica Ultracut E ultramicrotome (Leica Microsystems, Wetzlar, Germany), and sections were placed on formvar and carbon-coated grids (Electron Microscopy Sciences). Sections of 70 nm thickness from the midsection of the tendon were cut perpendicular to the longitudinal axis of the tendon. The sections were contrast stained with 2% uranyl acetate followed by lead citrate (Sigma-Aldrich) and imaged in a JEOL 1400 transmission electron microscope (JEOL, Peabody, MA, United States) equipped with a Gatan Orius SC1000 digital CCD camera (Gatan, Pleasanton, CA, United States).

Four regions of interest (ROI) were identified at ×2,500 magnification in each quadrant of the sample grid. Ten micrographs per sample from five regions of interest within the tendon were chosen from four quadrants and the center of the transverse section of the tissue, taken at a final magnification of ×25,000.

### 2.6 Collagen fibril image analysis

The ten representative images from each sample were preprocessed and quantified for collagen morphological parameters using the freely available Fiji image-processing package (Rigozzi et al., 2010). The quantified measurements from these ten images were averaged to provide a representative value for each sample. To correct for uneven backgrounds, the original images were subtracted by their Gaussian filtered (radius = 40 pixels) image to generate a new image with a “flat field” (Zhou et al., 2013). A median filter (radius = 5 pixels) was applied, followed by auto-thresholding to generate a binary mask. Outliers smaller than 5 pixels were removed. The resulting binary images were subjected to watershed segmentation and particle analysis to quantify each collagen fibril’s perimeter, area, radius, and total collagen area fraction. Fibrils on the edges of the images were excluded to remove samples that did not provide a complete characterization for quantification. These measurements described collagen morphological properties (collagen area fraction and collagen fibril radius) and a fibril-fibril interaction parameter (specific fibril surface) (Rigozzi et al., 2010). The distribution of fibril radii was presented in a histogram.

### 2.7 Quantitative gene expression

Following euthanasia, the loaded Achilles tendons were dissected free of soft tissue, as described previously, and harvested and stored in 1 mL of TRIzol reagent (Invitrogen, Waltham, MA, United States), fast frozen with liquid nitrogen, and stored at −80°C for further processing.

Total RNA from all tissue types was extracted from an established methodology. Tendon tissue was homogenized in 1 mL TRIzol with a Polytron homogenizer (850 Homogenizer, ThermoFisher Scientific, Waltham, MA, United States). RNA extraction and purification were performed using a PureLink RNA mini kit (Invitrogen, Waltham, MA, United States) per manufacturer instructions. A spectrophotometer (NanoDrop Technologies, Wilmington, DE, United States) was used to determine RNA concentrations and purity ratios.

For cDNA synthesis, 500 ng RNA for each sample was converted into cDNA using the PrimeScript RT reagent Kit with gDNA Eraser (TaKara Bio, Kusatsu, Shiga, Japan) according to the manufacturer’s protocol. SYBR Green-based quantitative PCR (qPCR) detection was then performed using PerfeCTA SYBR Green FastMix, Low ROX (Qiagen, Hilden, Germany) on a Mx3000P QPCR System (Stratagen, La Jolla, CA, United States). For each target mRNA, 2 µL diluted cDNA was amplified in an 11 µL SYBR Green PCR total reaction containing 5 µL SYBR Green and 0.6 µL (10 µM) of the forward and reverse primer. Samples were assessed for gene expression of matrix proteins (collagen types 1, 3, decorin, biglycan), matrix metalloproteinases (MMP-2, MMP-3, MMP-13), tissue inhibitors of matrix metalloproteinases (TIMP-1, TIMP-2), inflammatory cytokines (IL-1β, VEGF, TGFβ, TNFα), tenocyte markers (scleraxis, tenomodulin), and mechanotranducers (YAP and TAZ) (Table 1). All primers were designed to span an intron. Gene expression values were normalized to the housekeeping gene, *GAPDH*, and the control group. Gene expression values were normalized to the housekeeping gene, GAPDH, and the control group], and the data was analyzed with the 2^−δδ*C*_T_^ values (Livak and Schmittengen, 2001). At the end of each RT-qPCR run, the melting curves were checked to confirm a single product disassociation point and technical replicates were averaged if Ct values were within a tight range.

**TABLE 1:** Forward and reverse gene sequences for qPCR.

Gene	Forward Sequence (5′ → 3′)	Reverse Sequence (5′ → 3′)
*GAPDH*	ACC​CCT​CCT​GGG​TTT​GTA​GT	CAT​CCA​AGC​ATT​CAA​CCG​GC
*Col1a1*	GCG​AAG​GCA​ACA​GTC​GAT​TC	GGA​CCT​GGT​CTG​GGG​ATA​CT
*Col3a1*	GCC​TAC​ATG​GAT​CAG​GCC​AA	CAT​GGC​CTT​GCG​TGT​TTG​AT
*DCN*	CGG​TGG​CAA​ATA​CCC​GGA​TTA	AGG​GGA​TTG​TCA​GGG​TCG​TA
*BGN*	GAC​AAA​CCG​ACA​GCC​TGA​CA	ATG​AGC​AGC​CCA​TCA​TCC​AA
*MMP2*	AGT​TGG​CCA​CAT​CTG​GTT​G	TTT​GGC​AGA​AGT​TGG​GGT​CAT
*MMP3*	GGT​GGA​TGC​TGT​CTT​TGA​AGC	CTC​CAT​GAA​AAG​ACT​CAG​AGG​A
*MMP13*	ACC​CAG​CCC​TAT​CCC​TTG​AT	TCT​CGG​GAT​GGA​TGC​TCG​TA
*TIMP1*	CCA​GGT​CCG​AGT​TGC​AGA​AA	TCC​TGA​GTC​TCC​CTA​GAG​CC
*IL-1β*	GCT​ACC​TAT​GTC​TTG​CCC​GT	TCA​CAC​ACT​AGC​AGG​TCG​TC
*TNFα*	ATC​GGT​CCC​AAC​AAG​GAG​GA	CGC​TTG​GTG​GTT​TGC​TAC​G
*VEGF*	ACG​ACA​GAA​GGG​GAG​CAG​AA	AGA​TGT​CCA​CCA​GGG​TCT​CA
*TGFβ*	CAG​AAC​CCC​CAT​TGC​TGT​CC	CAG​CCA​CTC​AGG​CGT​ATC​AG
*Scx*	AAC​AGA​TCT​GCA​CCT​TCT​GCC	CTT​CGA​ATC​GCC​GTC​TTT​CTG
*Tnmd*	AGA​CAA​GCA​AGC​GAG​GAA​GAC	CAC​GAC​AGA​TGA​CTC​GAC​CTC
*YAP1*	TTC​GGC​AGG​CAA​TAC​GGA​A	TGG​CTG​CGG​AGA​GCT​AAT​TC
*TAZ*	GTG​GGA​GAT​GAC​CTT​CAC​GG	CAA​GAT​TGG​GCT​GGG​ACA​CT

### 2.8 Statistical analysis

Statistical analysis used GraphPad (version 9.3.0 for Windows; GraphPad Software, San Diego, CA, United States). The ROUT method was used to remove any outliers before analysis. The Kolmogorov-Smirnov test was used to test for normality. A one-way analysis of variance (ANOVA) test was used for normally distributed data to test the significance between groups on the mechanical, gene expression, and morphological properties. Otherwise, the Kruskal-Wallis test was performed. Statistical significance was assessed at *p*-values less than 0.05.

## 3 Results

### 3.1 Decreased *in vivo* mechanical properties with successive loading

**FIGURE 2:**
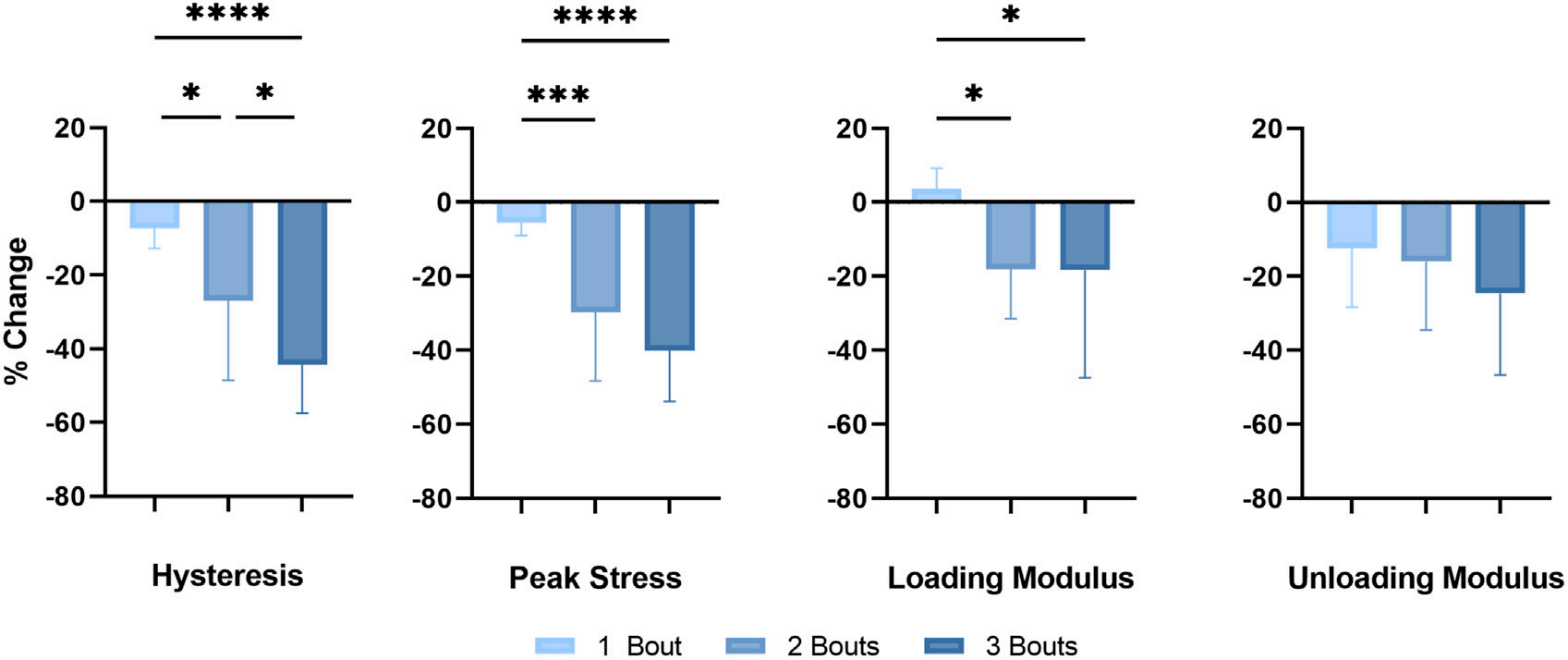
*In vivo* mechanical properties: Successive fatigue-load injuries from passive ankle dorsiflexion result in reduced mechanical properties, specifically in the percent change in hysteresis, the peak stress of each applied cycle, and the loading and unloading modulus between the uninjured *in vivo* tendon properties and properties following the final loading bout of each respective group. The data is presented as mean ± STD with statistical difference denoted by stars (*) between each number of load (* = *p* < 0.05, ** = *p* < 0.01, *** = *p* < 0.001, and **** = *p* < 0.0001).

Successive bouts of loading resulted in larger decreases in the *in vivo* mechanical properties of the fatigue-loaded Achilles tendons (Figure 2). There was a significant difference in hysteresis reduction, a mechanical property of viscoelastic tissues measuring their damping capacity, between the loading groups, with an even greater difference between groups 1 and 3. The decrease in peak stress and loading modulus of the dorsiflexion cycle was significant between group 1 and groups 2 and 3; however, there were no differences in the unloading moduli between the loading groups.

### 3.2 Differential gene expression in matrix and inflammatory markers with successive loads

**FIGURE 3:**
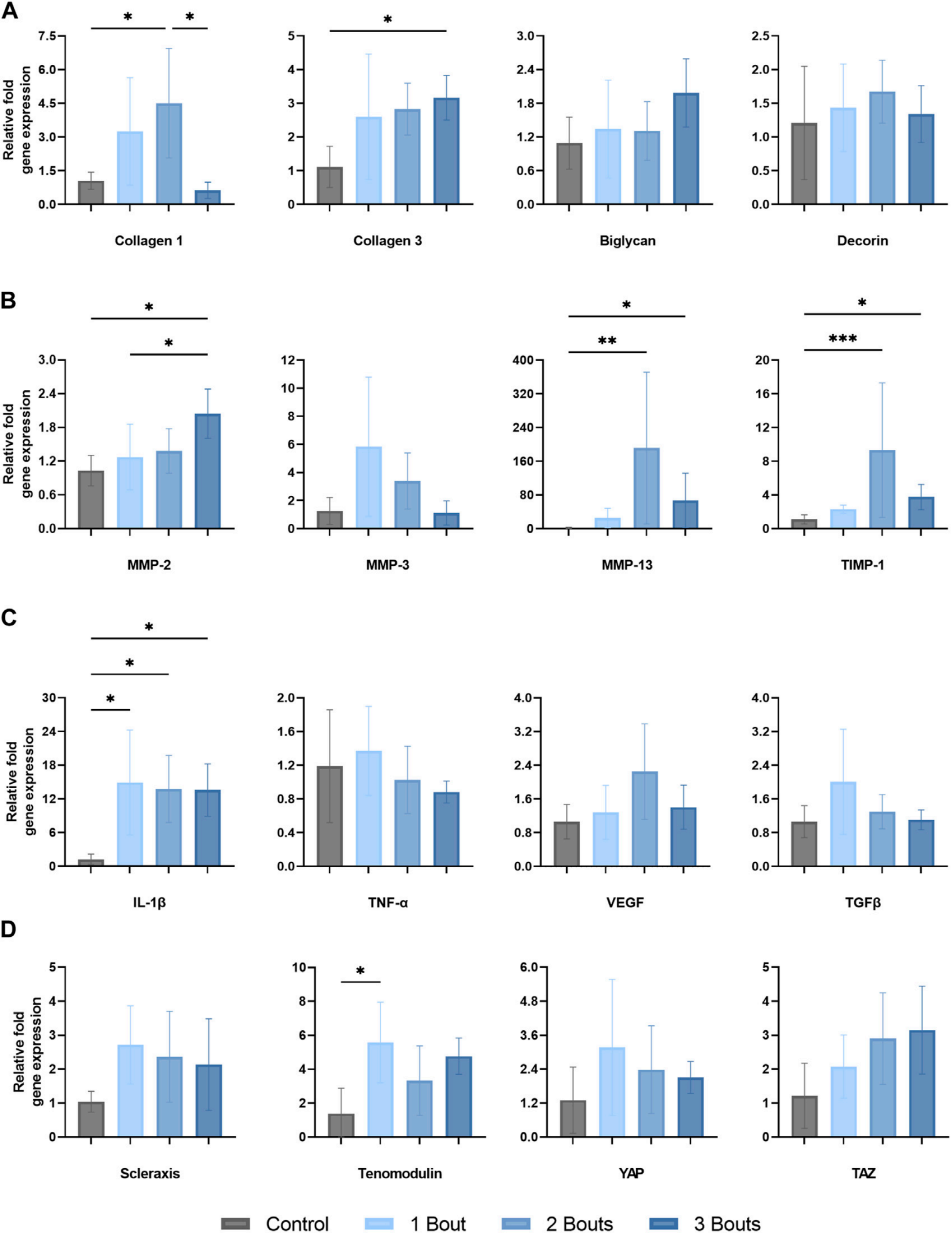
Gene expression levels of Achilles tendon mRNA relative to GAPDH of **(A)** matrix proteins (type I and III collagen, biglycan, and decorin, **(B)** matrix metalloproteinases and tissue inhibitor-1 (MMP-2, MMP-3, MMP-13, TIMP-1), **(C)** proinflammatory (IL-1B, TNF-a, and VEGF) and anti-inflammatory (TGFB) cytokines and (D) tenocyte markers (scleraxis, tenomodulin), and mechanotranducers (YAP and TAZ). The data is presented as mean ± STD with statistical difference denoted by stars (*) between each number of load (* = *p* < 0.05, ** = *p* < 0.01, and *** = *p* < 0.001).

There was an increase in type I collagen gene expression in groups 1 and 2, with a significant increase in group 2 compared to control (Figure 3A). There was a significant reduction in type I collagen gene expression, while there was an increase in type III collagen gene expression in group 3. However, there were no significant changes in the expression of biglycan and decorin, small leucine-rich proteoglycans (SLRPs) responsible for regulating the tendon’s collagen fibrillogenesis and extracellular matrix assembly.

There was an approximately 200-fold relative upregulation of MMP-13 in group 2, followed by a decrease in group 3, while still significantly upregulated around 65-fold compared to the control group (Figure 3B). MMP-2 expression reached significance in group 3 compared to groups 1 and control. The corresponding tissue inhibitor TIMP-1 was upregulated by 9.3-fold in group 2, followed by a decrease to 3.75-fold upregulation in group 3. MMP-3 expression had an increasing trend in group 1, followed by reductions in expression levels in subsequent groups, with no significance.

The pro-inflammatory cytokine IL-1β exhibited an average increase of roughly 14 to 15-fold across all three groups (Figure 3C). TNF-α, VEGF, and TGFβ did not change significantly throughout the loading groups. However, there was a slight upregulation for VEGF in group 2 and for TGFβ in group 1; however, neither reached significance.

### 3.3 Alterations in tenocyte and mechanotransduction markers

Scleraxis (Scx) had a 2.7-fold increase in gene expression following 1 bout of loading, with a slight decline for the remaining two groups, while still upregulated around 2-fold. Tenomodulin (Tnmd) was significantly upregulated by 5.6-fold for group 1 compared to the control group while staying relatively upregulated for the other groups. The mechanoresponsive transcriptional co-activators, Yes-Associated Protein (YAP) and Transcriptional co-activator with PDZ binding motif (TAZ), co-regulators of cellular proliferation and differentiation, had opposite trends between groups. While YAP expression increased 3.1-fold for group 1 and had a decreasing trend for the remaining groups, TAZ initially was upregulated by 2.1-fold and continued to increase (Figure 3D).

### 3.4 Cellular damage indicated early apoptotic processes

**FIGURE 4:**
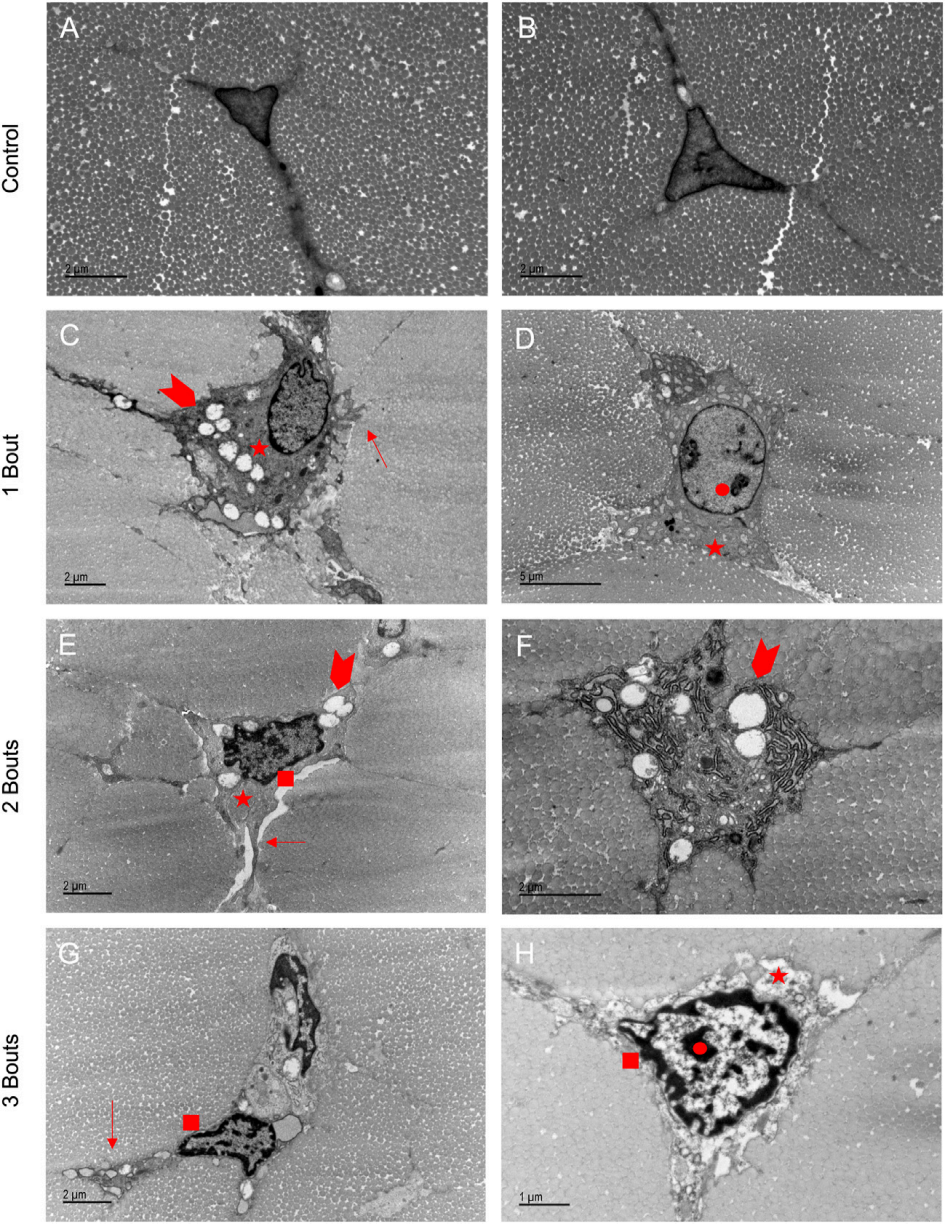
Loaded groups exhibited characteristics of apoptosis in TEM images: Healthy tenocytes exhibited characteristic elongated shapes with extensions into the matrix **(A, B)**. Loaded cells had accumulations of vesicles, vacuoles, and mitochondria (arrowheads) **(C, E, F)**. The shapes became more rounded with protrusions in the boundaries (arrows) **(C, E, G)**. Thickened pericellular matrix separated the cell from surrounding fibrils (stars) **(C, D, E, H)**. Some cells had dilated endoplasmic reticulum **(F)**. Apoptotic processes were demonstrated by areas of dense chromatin (circle) (D,H) and rings of chromatin around the cell boundary (square) **(E, G, H)**, with some cells in group 3 having separated nuclei **(G)**.

Tenocytes in the control group had the characteristic elongated cell shape with cell extensions into the network of collagen fibrils (Figures 4A, B). With fatigue overloading, cells became malformed and rounded, with accumulations of intracellular vacuoles, vesicles, and mitochondria (Figures 4C–H). The characteristic cytoplasm was preserved in the control groups, but with overload, there were disrupted cytoplasmic processes dominated by the nucleus, with some protrusions on the outer boundary of cells (Figures 4C–E, G, H). Healthy tenocytes contained uncondensed chromatin, and with successive bouts of loading, the chromatin started to form condensed regions (Figures 4D, E, H) and some developed rings of chromatin in the outer border of the cell (Figures 4C, G). In most tenocytes of the loaded groups, a thickened pericellular matrix of non-collagenous matrix formed, separating the cell from the surrounding fibrils. Damaged cells also exhibited dilated rough endoplasmic reticulum (Figure 4F). In group 3, tenocytes showed signs of apoptosis with nuclei fragmentation (Figure 4G).

### 3.5 Macro- and micro-structure indicates cellular and fibril adaptation response

**FIGURE 5:**
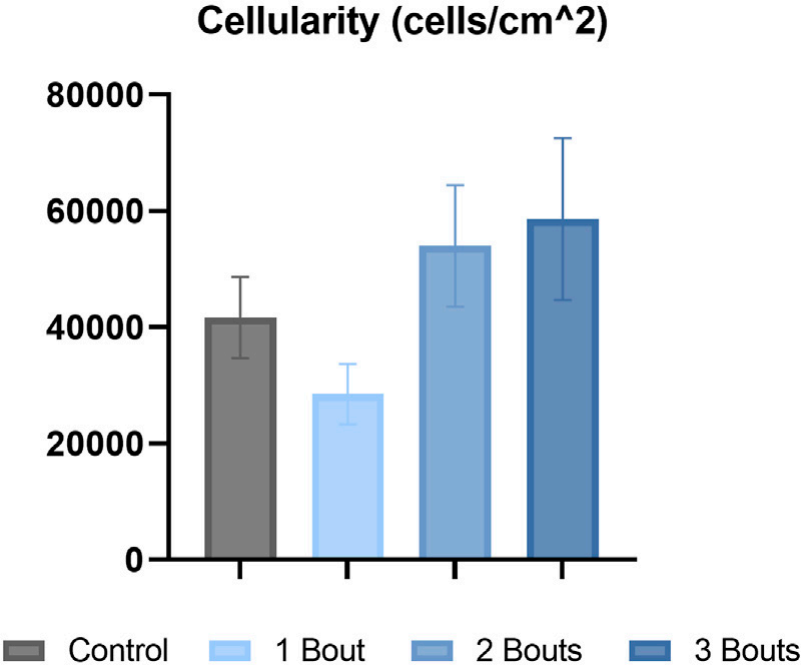
Hematoxylin & eosin (H&E) stained images were analyzed for cellularity for each group.

**FIGURE 6:**
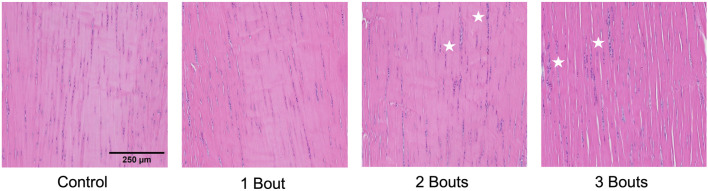
Uninjured and injured (groups 1, 2, and 3) Achilles tendon with hematoxylin and eosin staining. Stars denote areas of dense cellularity.

**FIGURE 7:**
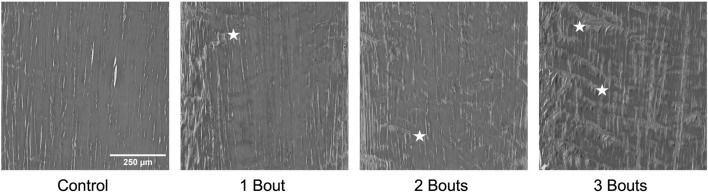
Representative images of collagen fiber structure from Masson’s Trichrome stained images in greyscale demonstrating the progressive increase in accumulation of fiber kinks denoted by stars. Panel 1 has no kinks, panel 2 and 3 show small areas of kinks, while panel 4 has a larger amount fibril kinking.

The control group’s nuclei had an average circularity of 0.5, slightly increasing to 0.55 for the groups with loading bouts. Similarly, the nuclear aspect ratio between the major and minor axes increased by approximately 10% between the control and the experimental groups. Both area fraction and cellularity decreased for group 1 compared to the control group and subsequently increased for groups 2 and 3 (Figure 5). Qualitatively, groups 2 and 3 exhibited areas of dense cellularity, and each group demonstrated a progressive increase in areas of fiber kinks (Figures 6, 7). The collagen orientation’s circular variance averaged 0.15 for all groups, with a subtle decrease between consecutive loading bouts and an average angle 45° angle. Neither of these structural properties showed statistical significance.

**FIGURE 8:**
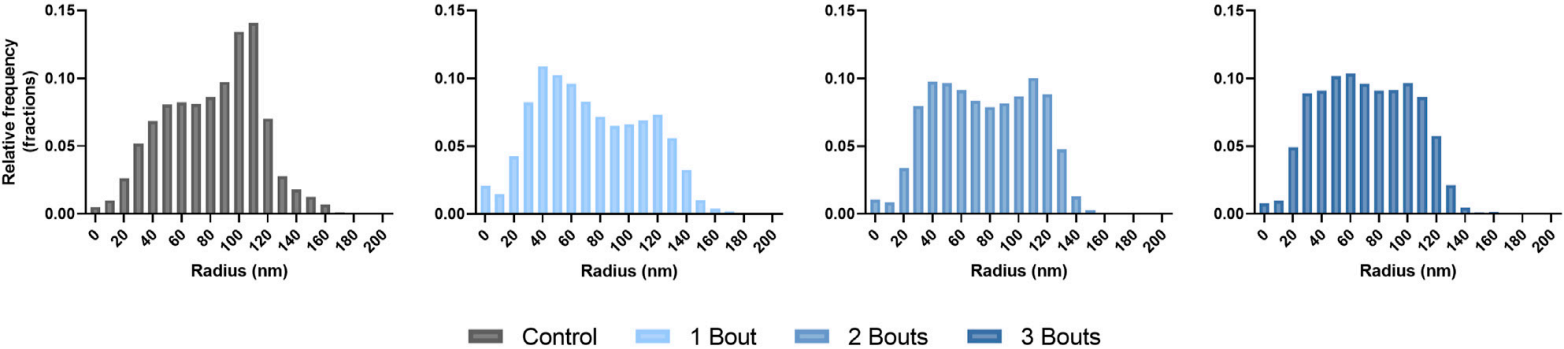
Histograms of collagen fibril diameter distributions presented in terms of fractional relative frequency of radii.

The histograms showed that the control group had a wide distribution of collagen fibrils with larger diameters. In contrast, group 1 had fewer large-diameter fibrils and more small-diameter ones. As the number of loads increased, the distribution began to normalize (Figure 8). The collagen area fraction stayed consistent throughout the groups, while the mean minor fibril radius and specific fibril surface decreased for the loaded groups compared to the control group (Table 2).

**TABLE 2:** Collagen morphological properties (collagen area fraction and fibril minor radius) and a fibril-fibril interaction parameter (specific fibril surface) for *n* = 1 sample for the control and loading groups.

Loading Group	Area Fraction (%)	Minor Radius (nm)	Specific Fibril Surface (µm µm^−2^)
0	78.37	83.53	0.0124
1	81.90	73.30	0.0117
2	81.70	75.35	0.0116
3	80.99	70.72	0.0110

## 4 Discussion

While previous animal models have mainly focused on long-term effects in tendinopathy due to overuse, our study offers novel insights by examining the immediate functional and mechanobiological responses to repeated fatigue loading. We accomplished this by conducting qualitative and quantitative assessments of mechanical properties, gene expression, and multi-scale tendon structure in rat Achilles tendons subjected to sub-failure fatigue loading and subsequent healing. Our findings demonstrate that all loading regimens resulted in significant reductions in hysteresis, peak stress, loading, and unloading moduli, along with notable alterations in the expression of inflammation and matrix turnover markers. Furthermore, the loading regimens caused both micro- and macro-structural damage. This research provides a unique perspective on the immediate impacts of overuse injuries, shedding light on the complex interplay of mechanical, mechanobiological, and structural changes in tendons during the acute healing phase.

The continual reduction in mechanical properties following each loading group suggests an accumulation of fatigue damage from successive loading regimens. The hysteresis loss with increased loading regimens is consistent with studies of *in vivo* fatigue loading of the rat patellar tendon (Andarawis-Puri et al., 2012). The changes in hysteresis, a viscoelastic property quantifying energy loss and damping capacity during tissue loading, may suggest alterations to the non-collagenous components in the extracellular matrix, loss in fibril crimp, and a reduced ability for the tendon to protect itself from further damage (Williamson et al., 2021). While our results showed reduced loading and unloading moduli following repetitive loading regimens, another study demonstrated an initial stiffness reduction followed by increased stiffness with further loading. The consistent reduction in moduli may result from continuous damage where only fibers in high tension are loaded, and subsequent decreases can be due to a lack of recovery for the fibers not in tension and ruptured collagen fibers that were previously loaded. The reduction in peak stress following fatigue loading suggests the tendon’s impaired ability to bear further stress and load redistribution from damaged to undamaged collagen fibers.

The successive mechanical stimuli caused alterations in mechanotransduction markers. YAP and TAZ are mechanotranducers of the Hippo signaling pathway that are considered to be activated by ECM stiffness and cell shape, whose activity enables cell responses to mechanical cues (Lavagnino et al., 2015). Their activity is required for fibroblast proliferation, and a stiffer ECM can activate a positive fibrotic feedback loop, maintaining or even activating injury states. These transcription factors also regulate other proteins responsible for matrix stiffening and cell contractility, supporting collagen remodeling. However, these transducers’ are inhibited upon cell detachment and with a rounder cell morphology (Dupont, 2016; Cai et al., 2021). In our study, YAP and TAZ were both upregulated for group 1. Interestingly, although these are co-transducers, our study showed a gradual decrease in YAP expression with increased TAZ expression for groups 2 and 3. The initial increase in YAP/TAZ may respond to the initial increase in matrix stiffness, as seen with the increase in loading modulus in group 1. With the initiation of apoptotic processes and decreased tendon stiffness, YAP gene expression gradually decreased between groups. While TAZ continued to increase, this may be due to the acute healing time point and a lag in the co-activators response. YAP and TAZ are considered to be regulated at the protein level by phosphorylation events. In this study, their changes are reported on the transcriptional level. YAP/TAZ regulation which can be considered to be due to upstream signalling pathways in the Hippo pathway, such as focal adhesion sensing of the ECM and adherens junctions mediating cellular adhesion (Heng et al., 2021).

Scleraxis, a transcription factor, is crucial for tenocyte differentiation and mechanically stimulated adult tendon growth. Scleraxis expression is known to positively regulate tenomodulin, a type II transmembrane glycoprotein, and a tendon-specific maturation marker, in a tendon cell lineage-dependent manner (Shukunami et al., 2006; Shukunami et al., 2018; Liu et al., 2021). Our findings show increased Scx and Tnmd expression across all groups, indicating tenocyte differentiation processes. The decreasing trend in expression between groups for both genes may indicate a loss in tenocyte differentiation and cell maturation into aberrant tissues. The increase in Tnmd expression between groups 2 and 3 may demonstrate an attempt to recover tenocyte maturation.

Recent work has hypothesized that inflammatory responses from overuse tendon injuries may precede tendon degeneration and pain (D’Addona et al., 2017). The initial responses to tendon injuries involve an inflammatory phase lasting 24 h to 1 week. This involves the migration and recruitment of inflammatory cells, which induces pro-inflammatory cytokines, such as IL-1ß, TNFα, VEGF, and growth factors, including TGFß (D'Addona et al., 2017). While our results show no significant difference in the expression of TNFα, VEGF, and TGFß, the expression of IL-1ß was consistently elevated following each loading group. Changes in cytokine levels alter the expression of type I and III collagen and matrix metalloproteinases (MMPs), which are involved in the remodeling and degradation of the extracellular matrix. Tendon remodeling is mediated by the balance of MMPs and their tissue inhibitors (TIMPs) (Del Buono et al., 2013). An imbalance between these two types of enzymes can disrupt homeostasis and collagen formation, contributing to the degenerative process leading to tendinopathy. IL-1ß induces degenerative tendon changes, including expression of MMP-1, -3, -8, and -13 and downregulation of collagen type I expression (D’Addona et al., 2017). MMP-13 is associated with matrix degradation, specifically the disruption of type I and III collagen in the early phases of wound healing. The significant increase in MMP-13 expression may signify an imbalance in the matrix remodeling process. The gradual increase in MMP-2 expression, which degrades smaller and denatured collagen fragments, demonstrates an active degradation of damaged collagen fibers from repetitive loading.

Our results of a decreased type I collagen expression after three loading bouts with an increase in type III collagen expression may indicate early signs of an impaired wound healing response and contribute to the decreased mechanical properties seen following fatigue loading. Increased collagen disorganization has been reported to precede symptoms of tendon injury and tendon rupture in previous studies (Docking et al., 2015), where alterations to collagen synthesis have been shown as early as 24 h post-loading (Magnusson et al., 2010), with increases in type I and III collagen, and the proportion of type III to I collagen. In the early healing stage, type III collagen is rapidly produced and irregularly placed as a “patch” to repair the damaged area (Maffulli et al., 2000). While type I collagen is eventually produced in normal tendons to replace type III collagen, tendinopathic tendon’s impaired healing mechanisms lead to increased production and accumulation of type III collagen (Millar et al., 2021). While the deposition of type III collagen is a rapid healing response to successive overloading, its disorganized arrangement contributes to the tendon’s inferior mechanics, predisposing the tendon to tendon ruptures (Maffulli et al., 2000). Our study showed a gradual increase in type I collagen expression for groups 1 and 2, followed by a sharp decrease for group 3. Meanwhile, there was a consistently elevated type III collagen expression between experimental groups. The shift in type I collagen expression with a consistent upregulation of type III collagen indicates the tendons’ response to produce type III collagen in recovery from successive injuries.

Cellular morphology became disrupted in loaded groups before the widespread appearance of collagen fiber kinking. While healthy tenocytes had cell extensions, demonstrating deep cell-matrix connections, the thickened pericellular matrix indicated broken cell-cell and cell-matrix interactions. The presence of vacuoles, vesicles, and mitochondria features confirms increased metabolic processes. The cellular damage is consistent with features seen in an *in situ* tendon fatigue model and human tendinopathic samples (Zabrzynski et al., 2018; Ros et al., 2019). Tenocytes exhibited features of apoptosis processes, forming dense chromatin regions, followed by a ring on the cell’s outer border and separation of nuclei. There was no formation of a “beaded necklace” or release of apoptotic bodies, as seen in the cell death process. This may correspond to samples being imaged at an early time point following healing. This early evidence of apoptosis indicates a disruption in cellular homeostasis, potentially interrupting the cellular signaling necessary for reparative processes and mechanotransduction and leading to downstream inflammation and imbalance in matrix turnover. As seen in our results, IL-1ß and MMP-2, two genes involved in the positive regulation of apoptotic processes, were upregulated, indicating a feedback mechanism in which apoptotic processes may begin in overloaded tendons (Van Opdenbosch and Lamkanfi, 2019; Kim et al., 2021). Apoptosis has been suggested to play a role in tendon overuse injuries, as demonstrated in clinical tendinopathy and other preclinical overuse models of tendinopathy (Scott et al., 2007; Millar et al., 2009; Ros et al., 2019).

There were no significant differences in collagen orientation, cellular morphology, and proliferation macro-structurally. However, qualitative changes included increased collagen fiber crimping and cellular proliferation in various tendon regions. Low cellularity levels detected within group 1 with respect to the control group could be attributed to cell death. In contrast, the subsequent increase in cellularity for groups 2 and 3 could indicate an increase in cellular response to injury. While there were trends among experimental groups, there was no significance, probably due to the low sample size. The increase in cell number can be attributed to IL-1ß, Scx, and YAP, all upregulated in all groups. Along with these macro-structural damage accumulations, the reduced expression of type I collagen and increased production of mechanically weaker type III collagen may result in the tendon experiencing lower mechanical and material properties (Arya and Kulig, 2010). On the fibrillar level, the increase in lower-diameter fibrils may indicate a microscale adaptation response, where collagen fibrils gradually grow following previous injuries. Since this study assessed early responses to successive fatigue injuries, there may be a lag in the appearance of macro-structural damage, as mechanobiological changes precede gross tissue changes.

While this study demonstrated the ability to use an *in vivo* overuse passive loading model to induce accumulated tendon damage, it is essential to acknowledge certain limitations associated with the work. Although we quantified *in vivo* functional changes resulting from successive loading bouts, it is important to note that comparisons between loading groups to assess healing were not feasible due to the nature of the prescribed fatigue loading regimen. This regimen was tailored to each animal based on the unique properties of their Achilles tendon loading-unloading curves rather than using a load- or displacement-controlled approach. The variability in the gene expression levels may have been attributed to the subject-specific loading protocol and the nature of *in vivo* experiments. Future studies employing load- or displacement-control modes could provide more precise quantifications of alterations during the healing period. While our *in vivo* mechanical measurements contributed to our understanding of the effects of the fatigue loading protocol, additional insights can be gained from traditional viscoelastic, dynamic, and failure tests and provide a comprehensive view of changes in tendon material properties. Our conversions from ankle dorsiflexion angle to strain rely on *ex vivo* measured strain values, and future work will explore non-invasive methods for quantifying *in vivo* tendon strains throughout loading using ultrasound. Tendon structure was quantified only at the acute healing time points. Investigating tendon structure under varying loading conditions can provide valuable insights into dynamic changes in collagen structure and damage. While our study reported gene expression of collagen, matrix, and inflammatory markers, the inclusion of other damage markers, including apoptosis-related genes, can enhance our understanding of the degenerative and remodeling response following fatigue injuries, given that TEM imaging demonstrated processes preceding apoptosis. Tendons comprise collagen, glycosaminoglycans, and water, collectively contributing to their viscoelastic material properties. A deeper understanding of changes in tendon composition and production can be achieved through assays that quantify the content and synthesis of these key tendon components. While this study focused on assessing tendon changes during the acute healing period, it is crucial to recognize that the inflammatory phase lasts up to 1 week. Future studies will aim to comprehensively characterize the alterations in tendon mechanobiological responses and functional changes in response to fatigue injuries.

We demonstrated early mechanical, biological, and structural damage to successive fatigue loading injuries from our novel passive loading system. This is the first animal model that establishes a direct connection between successive *in vivo* fatigue loads and mechanobiological and functional tendon changes. Our findings demonstrate the early, adaptive responses of tendons to overloading and show promise for longer-term investigations into the underlying mechanisms of cellular signaling and damage and matrix turnover in response to *in vivo* injuries caused by overuse and aging to enhance our ability to predict and address early-stage tendinopathy, ultimately improving the prognosis for individuals facing such conditions.

## Data Availability

The raw data supporting the conclusion of this article will be made available by the authors, without undue reservation.

## References

[B1] Andarawis-Puri N. Flatow E. L. (2011). Tendon fatigue in response to mechanical loading. J. Musculoskelet. Neuronal Interact. 11, 106–114. 21625047 PMC4408766

[B2] Andarawis-Puri N. Philip A. Laudier D. Schaffler M. B. Flatow E. L. (2014). Temporal effect of *in vivo* tendon fatigue loading on the apoptotic response explained in the context of number of fatigue loading cycles and initial damage parameters. J. Orthop. Res. 32, 1097–1103. 10.1002/jor.22639 24838769 PMC4209741

[B3] Andarawis-Puri N. Sereysky J. B. Jepsen K. J. Flatow E. L. (2012). The relationships between cyclic fatigue loading, changes in initial mechanical properties, and the *in vivo* temporal mechanical response of the rat patellar tendon. J. Biomech. 45, 59–65. 10.1016/j.jbiomech.2011.10.008 22055428 PMC3763928

[B4] Arya S. Kulig K. (2010). Tendinopathy alters mechanical and material properties of the Achilles tendon. J. Appl. Physiol. (1985) 108, 670–675. 10.1152/japplphysiol.00259.2009 19892931

[B5] Benage L. G. Sweeney J. D. Giers M. B. Balasubramanian R. (2022). Dynamic load model systems of tendon inflammation and mechanobiology. Front. Bioeng. Biotechnol. 10, 896336. 10.3389/fbioe.2022.896336 35910030 PMC9335371

[B6] Bloom E. T. Lin L. M. Locke R. C. Giordani A. Krassan E. Peloquin J. M. (2023). Overload in a rat *in vivo* model of synergist ablation induces tendon multiscale structural and functional degeneration. J. Biomech. Eng. 145, 081003. 10.1115/1.4062523 37184932 PMC10782872

[B7] Cai X. Wang K. C. Meng Z. (2021). Mechanoregulation of YAP and TAZ in cellular homeostasis and disease progression. Front. Cell Dev. Biol. 9, 673599. 10.3389/fcell.2021.673599 34109179 PMC8182050

[B49] Chainani P. H. Williamson P. M. Yeritsyan D. Momenzadeh K. Kheir N. DeAngelis J. P. (2024). A passive ankle dorsiflexion testing system for an In Vivo model of overuse-induced tendinopathy. 10.3791/65803 38497634

[B8] D'addona A. Maffulli N. Formisano S. Rosa D. (2017). Inflammation in tendinopathy. Surgeon 15, 297–302. 10.1016/j.surge.2017.04.004 28596062

[B9] Del Buono A. Oliva F. Osti L. Maffulli N. (2013). Metalloproteases and tendinopathy. Muscles Ligaments Tendons J. 3, 51–57. 10.11138/mltj/2013.3.1.051 23885345 PMC3676164

[B10] Docking S. I. Ooi C. C. Connell D. (2015). Tendinopathy: is imaging telling us the entire story? J. Orthop. Sports Phys. Ther. 45, 842–852. 10.2519/jospt.2015.5880 26390270

[B11] Dupont S. (2016). Role of YAP/TAZ in cell-matrix adhesion-mediated signalling and mechanotransduction. Exp. Cell Res. 343, 42–53. 10.1016/j.yexcr.2015.10.034 26524510

[B12] Fung D. T. Wang V. M. Laudier D. M. Shine J. H. Basta-Pljakic J. Jepsen K. J. (2009). Subrupture tendon fatigue damage. J. Orthop. Res. 27, 264–273. 10.1002/jor.20722 18683881 PMC4786739

[B13] Helland C. Bojsen-Moller J. Raastad T. Seynnes O. R. Moltubakk M. M. Jakobsen V. (2013). Mechanical properties of the patellar tendon in elite volleyball players with and without patellar tendinopathy. Br. J. Sports Med. 47, 862–868. 10.1136/bjsports-2013-092275 23833044

[B48] Heng B. C. Zhang X. Aubel D. Bai Y. Li X. Wei Y. (2021). An overview of signaling pathways regulating YAP/TAZ activity. Cell Mol. Life Sci. 78 (2), 497–512. 10.1007/s00018-020-03579-8 32748155 PMC11071991

[B14] Kaux J. F. Forthomme B. Goff C. L. Crielaard J. M. Croisier J. L. (2011). Current opinions on tendinopathy. J. Sports Sci. Med. 10, 238–253. 24149868 PMC3761855

[B15] Kim R. J. An S. H. Gwark J. Y. Park H. B. (2021). Antioxidant effects on hypoxia-induced oxidative stress and apoptosis in rat rotator cuff fibroblasts. Eur. Cell Mater 41, 680–693. 10.22203/ecm.v041a44 34114203

[B16] Komi P. V. Fukashiro S. Jarvinen M. (1992). Biomechanical loading of Achilles tendon during normal locomotion. Clin. Sports Med. 11, 521–531. 10.1016/s0278-5919(20)30506-8 1638639

[B17] Lavagnino M. Wall M. E. Little D. Banes A. J. Guilak F. Arnoczky S. P. (2015). Tendon mechanobiology: current knowledge and future research opportunities. J. Orthop. Res. 33, 813–822. 10.1002/jor.22871 25763779 PMC4524513

[B18] Lipman K. Wang C. Ting K. Soo C. Zheng Z. (2018). Tendinopathy: injury, repair, and current exploration. Drug Des. Devel Ther. 12, 591–603. 10.2147/dddt.s154660 PMC5865563 29593382

[B19] Liu H. Xu J. Lan Y. Lim H. W. Jiang R. (2021). The scleraxis transcription factor directly regulates multiple distinct molecular and cellular processes during early tendon cell differentiation. Front. Cell Dev. Biol. 9, 654397. 10.3389/fcell.2021.654397 34150754 PMC8211106

[B47] Livak K. J. Schmittgen T. D. (2001). Analysis of relative gene expression data using real-time quantitative PCR and the 2^−δδ*C* _T_ ^ method. Methods 25 (4), 402–408. 10.1006/meth.2001.1262 11846609

[B20] Maffulli N. Ewen S. W. Waterston S. W. Reaper J. Barrass V. (2000). Tenocytes from ruptured and tendinopathic achilles tendons produce greater quantities of type III collagen than tenocytes from normal achilles tendons. An *in vitro* model of human tendon healing. Am. J. Sports Med. 28, 499–505. 10.1177/03635465000280040901 10921640

[B21] Maffulli N. Longo U. G. Kadakia A. Spiezia F. (2020). Achilles tendinopathy. Foot Ankle Surg. 26, 240–249. 10.1016/j.fas.2019.03.009 31031150

[B22] Magnusson S. P. Langberg H. Kjaer M. (2010). The pathogenesis of tendinopathy: balancing the response to loading. Nat. Rev. Rheumatol. 6, 262–268. 10.1038/nrrheum.2010.43 20308995

[B23] Millar N. L. Silbernagel K. G. Thorborg K. Kirwan P. D. Galatz L. M. Abrams G. D. (2021). Tendinopathy. Nat. Rev. Dis. Prim. 7, 1. 10.1038/s41572-020-00234-1 33414454

[B24] Millar N. L. Wei A. Q. Molloy T. J. Bonar F. Murrell G. A. (2009). Cytokines and apoptosis in supraspinatus tendinopathy. J. Bone Jt. Surg. Br. 91, 417–424. 10.1302/0301-620x.91b3.21652 19258623

[B25] Neviaser A. Andarawis-Puri N. Flatow E. (2012). Basic mechanisms of tendon fatigue damage. J. Shoulder Elb. Surg. 21, 158–163. 10.1016/j.jse.2011.11.014 PMC3749775 22244058

[B26] Ng G. Y. Chung P. Y. Wang J. S. Cheung R. T. (2011). Enforced bipedal downhill running induces Achilles tendinosis in rats. Connect. Tissue Res. 52, 466–471. 10.3109/03008207.2011.562334 21591929

[B27] Pentzold S. Wildemann B. (2022). Mechanical overload decreases tenogenic differentiation compared to physiological load in bioartificial tendons. J. Biol. Eng. 16, 5. 10.1186/s13036-022-00283-y 35241113 PMC8896085

[B28] Rigozzi S. Muller R. Snedeker J. G. (2010). Collagen fibril morphology and mechanical properties of the Achilles tendon in two inbred mouse strains. J. Anat. 216, 724–731. 10.1111/j.1469-7580.2010.01225.x 20345854 PMC2952385

[B29] Ros S. J. Muljadi P. M. Flatow E. L. Andarawis-Puri N. (2019). Multiscale mechanisms of tendon fatigue damage progression and severity are strain and cycle dependent. J. Biomech. 85, 148–156. 10.1016/j.jbiomech.2019.01.026 30732906 PMC6608713

[B30] Sarver D. C. Kharaz Y. A. Sugg K. B. Gumucio J. P. Comerford E. Mendias C. L. (2017). Sex differences in tendon structure and function. J. Orthop. Res. 35, 2117–2126. 10.1002/jor.23516 28071813 PMC5503813

[B31] Scott A. Cook J. L. Hart D. A. Walker D. C. Duronio V. Khan K. M. (2007). Tenocyte responses to mechanical loading *in vivo*: a role for local insulin-like growth factor 1 signaling in early tendinosis in rats. Arthritis Rheum. 56, 871–881. 10.1002/art.22426 17328060

[B32] Shukunami C. Takimoto A. Nishizaki Y. Yoshimoto Y. Tanaka S. Miura S. (2018). Scleraxis is a transcriptional activator that regulates the expression of Tenomodulin, a marker of mature tenocytes and ligamentocytes. Sci. Rep. 8, 3155. 10.1038/s41598-018-21194-3 29453333 PMC5816641

[B33] Shukunami C. Takimoto A. Oro M. Hiraki Y. (2006). Scleraxis positively regulates the expression of tenomodulin, a differentiation marker of tenocytes. Dev. Biol. 298, 234–247. 10.1016/j.ydbio.2006.06.036 16876153

[B34] Soslowsky L. J. Thomopoulos S. Esmail A. Flanagan C. L. Iannotti J. P. Williamson J. D. 3rd (2002). Rotator cuff tendinosis in an animal model: role of extrinsic and overuse factors. Ann. Biomed. Eng. 30, 1057–1063. 10.1114/1.1509765 12449766

[B35] Soslowsky L. J. Thomopoulos S. Tun S. Flanagan C. L. Keefer C. C. Mastaw J. (2000). Neer Award 1999. Overuse activity injures the supraspinatus tendon in an animal model: a histologic and biomechanical study. J. Shoulder Elb. Surg. 9, 79–84. 10.1016/s1058-2746(00)90033-8 10810684

[B36] Spiesz E. M. Thorpe C. T. Chaudhry S. Riley G. P. Birch H. L. Clegg P. D. (2015). Tendon extracellular matrix damage, degradation and inflammation in response to *in vitro* overload exercise. J. Orthop. Res. 33, 889–897. 10.1002/jor.22879 25721513 PMC4855636

[B37] Svensson R. B. Heinemeier K. M. Couppe C. Kjaer M. Magnusson S. P. (2016). Effect of aging and exercise on the tendon. J. Appl. Physiol. (1985) 121, 1237–1246. 10.1152/japplphysiol.00328.2016 27150831

[B38] Tarantino D. Mottola R. Resta G. Gnasso R. Palermi S. Corrado B. (2023). Achilles tendinopathy pathogenesis and management: a narrative review. Int. J. Environ. Res. Public Health 20, 6681. 10.3390/ijerph20176681 37681821 PMC10487940

[B39] Thorpe C. T. Chaudhry S. Lei I. I. Varone A. Riley G. P. Birch H. L. (2015). Tendon overload results in alterations in cell shape and increased markers of inflammation and matrix degradation. Scand. J. Med. Sci. Sports 25, e381–e391. 10.1111/sms.12333 25639911

[B40] Van Opdenbosch N. Lamkanfi M. (2019). Caspases in cell death, inflammation, and disease. Immunity 50, 1352–1364. 10.1016/j.immuni.2019.05.020 31216460 PMC6611727

[B41] Wiesinger H. P. Seynnes O. R. Kosters A. Muller E. Rieder F. (2020). Mechanical and material tendon properties in patients with proximal patellar tendinopathy. Front. Physiol. 11, 704. 10.3389/fphys.2020.00704 32733263 PMC7358637

[B42] Williamson P. M. Freedman B. R. Kwok N. Beeram I. Pennings J. Johnson J. (2021). Tendinopathy and tendon material response to load: what we can learn from small animal studies. Acta Biomater. 134, 43–56. 10.1016/j.actbio.2021.07.046 34325074 PMC8542586

[B43] Williamson P. M. Yeritsyan D. Peacock T. Chainani P. Momenzadeh K. Asciutto D. (2023). A passive ankle dorsiflexion testing system to assess mechanobiological and structural response to cyclic loading in rat Achilles tendon. J. Biomech. 156, 111664. 10.1016/j.jbiomech.2023.111664 37302164 PMC10439675

[B44] Zabrzynski J. Gagat M. Paczesny L. Lapaj L. Grzanka D. (2018). Electron microscope study of the advanced tendinopathy process of the long head of the biceps brachii tendon treated arthroscopically. Folia Morphol. Warsz. 77, 371–377. 10.5603/fm.a2017.0105 29131279

[B45] Zamboulis D. E. Thorpe C. T. Ashraf Kharaz Y. Birch H. L. Screen H. R. Clegg P. D. (2020). Postnatal mechanical loading drives adaptation of tissues primarily through modulation of the non-collagenous matrix. Elife 9, e58075. 10.7554/elife.58075 33063662 PMC7593091

[B46] Zhou Y. Hong W. Lu L. (2013). Imaging beads-retained prey assay for rapid and quantitative protein-protein interaction. PLoS One 8, e59727. 10.1371/journal.pone.0059727 23555762 PMC3612083

